# Prevalence of Squamous and Basal Cell Carcinomas in African Albino Skin Cancer Lesions: A Systematic Review and Meta-Analysis of Proportion

**DOI:** 10.1155/2022/5014610

**Published:** 2022-08-30

**Authors:** Nnaemeka T. Onyishi, Samuel R. Ohayi

**Affiliations:** Histopathology Department, Enugu State University College of Medicine Parklane, Enugu, Nigeria

## Abstract

**Objective:**

To estimate the prevalence of cutaneous SCC and BCC in all histologically confirmed skin cancer lesions in African albinos.The following five databases are as follows: African Journals Online (AJOL), PubMed, Europe PMC, and Google Scholar were searched for relevant articles. Study Selection: included studies were case series and cross-sectional studies of histologically confirmed skin cancers in African albinos. Data extraction and synthesis: data extraction and synthesis was informed by the meta-analysis of observational studies in epidemiology guideline. By random effect meta-analysis, we calculated the pooled prevalence of SCC and BCC in skin cancer lesions of the African albinos.

**Result:**

We abstracted 695 skin cancer lesions from 540 African albinos (275 male and 241 female albinos with sex not stated in 24 subjects). There were 419 SCCs and 249 BCCs. By meta-analysis, the pooled prevalence of SCC is 64% (95% CI; 50–77%). The prevalence for BCC is 31% (95% CI; 19–45%).

**Conclusion:**

Overall, squamous cell carcinoma is the predominant type of keratinocyte carcinoma reported in African albinos. SCC is preponderant in case series of surgical excision biopsies while BCC predominates in studies reporting on albino skin surveillance programmes.

## 1. Introduction

Keratinocyte carcinoma-squamous cell carcinoma (SCC) and basal cell carcinoma (BCC) are the most common malignant neoplasm in fair skinned populations [1]. It was estimated that over 5 million nonmelanoma skin cancers existed in the United States and that over 3 million people had been treated for nonmelanoma skin cancers in 2012 [2]. Also, the incidence rates for SCC and BCC have been found to be very high in parts of Australia and England [1, 3, 4].

In contrast, pigmented Africans have low risk for cutaneous malignancies. A comprehensive review estimates that skin cancer accounts for 20%–30% of all neoplasms in Caucasians but only 1%–2% in black people [5]. While the keratinocyte carcinomas and cutaneous melanomas seemingly account for about 40% of all malignant neoplasms in the US whites [6], a number of hospital studies in Africa disclose that skin cancers constitute only 5.5%–13% of all diagnosed malignancies [7–9].

However, a subpopulation of Africans, the African albinos, has elevated risk for skin cancer. Albinos tend to develop multiple cutaneous malignancies, at younger age and in sun-exposed body sites. This increased propensity for cutaneous malignancies derives from genetically inherited disorder in skin melanization, which bequeaths the albino with hypomelanized, sun-sensitive skin susceptible to cutaneous carcinogenesis [10].

Studies show that epidemiology and the incidence proportion of the keratinocyte carcinomas (SCC and BCC) differ in Caucasians and pigmented Africans. Caucasians have propensity for multiple keratinocyte carcinomas, which feature more at sun-exposed body sites, and with respect to the incidence proportion, there is a preponderance of BCC over SCC in Caucasians [11]. The BCC to SCC incidence ratio of about 4 : 1 had previously been reported, but recent studies suggest that this ratio narrows significantly with increasing age [2, 11, 12]. In contrast, pigmented Africans rarely develop BCC. In a recent analysis of 450 African patients with primary cutaneous malignancy in a Nigerian hospital, 39 had BCC and 74% (29/39) of these occurred in African albinos [13]. Most keratinocyte carcinomas in pigmented Africans are SCC carcinomas, often occurring in non-sun-exposed sites and arising from or commonly associated with chronic inflammatory conditions and scars [14].

The epidemiology of keratinocyte carcinomas in African albinos mirrors that of the Caucasians in some respect. Similar to the Caucasians, keratinocyte carcinomas in African albinos tend to be multifocal and more at sun-exposed body sites. [13] But, there have been variations in the incidence proportion of keratinocyte carcinomas reported in African albinos. While some studies [15] report a preponderance of SCC over BCC, similar to pigmented Africans. Some other studies [16, 17] have found BCC to be more frequent than SCC in African albinos consistent with the situation in Caucasians. With these reported discrepancies in view, we undertook a systematic review and meta-analysis of all existing studies reporting on skin cancers in African albinos aiming to establish the prevalence of the various types of keratinocyte carcinomas (SCC and BCC) among African albinos with skin cancer.

## 2. Methods

Methods adapted for this review had been previously described in our first manuscript, which focused on cutaneous melanoma [18].

### 2.1. Literature Search and Study Selection

Four indexing sites, considered the preferred hosts of the African biomedical literature, were comprehensively searched for eligible publications. The sites were African Journals Online (AJOL), Google Scholar, PubMed, and Europe PMC. The search was conducted on May 28, 2020, and updated on September 2020. The databases were searched iteratively with the following string of key terms: “skin cancer in African albinos,” “cutaneous malignancy in African albinos,” and “skin cancer in Africans.” As this is a second of a two-part report, the full-search strategy used in data acquisition has been detailed in our first publication that focused on melanoma skin cancers [18].

### 2.2. Inclusion and Exclusion Criteria

The included studies are case series or cross-sectional studies of skin cancer in African albinos. In the included studies, skin cancer lesions must have been histologically classified and African albinos were the overall subject of the study or were identified as a subgroup in a larger sample of Africans [18]. Excluded were all studies in which the malignant diagnosis was not histologically confirmed and all skin cancer cases in albino individuals not of African descent or of African descent but not reported from Sub-Saharan Africa [18].

### 2.3. Data Collection

Data extracted from the articles include author, year of study, country of study, type of study, brief description of study, total number of subjects, no of albinos with skin cancer, sex distribution of albinos with skin cancer, mean and median age of albinos with skin cancer, and histologic types of albino skin cancer. The process of data extraction was independently undertaken by the two authors and disagreements were resolved by discussion and consensus among the authors [18].

### 2.4. Quality Assessment of Individual Studies

Methodological quality of the included studies was assessed using a modification of Newcastle–Ottawa Scale adapted for case series [19]. This tool consists of eight items under four domains. Some of the items are related to reports of adverse drug event and thus are not relevant to determining the validity of studies included in our review. Our quality assessment was based on scores in the domain of selection, ascertainment, and reporting. Each of the included study scored one or two points in each of these three domains. An aggregate score of 3 or 4 was considered low quality while a score of 5 or 6 was appraised high quality. Discordant assessments were resolved by consensus among the authors (Table 1) [18].

### 2.5. Statistical Analysis

We used the metafor package of R statistical software to calculate the prevalence proportions of SCC and BCC in the aggregate skin cancer burden of African albinos. African albinos often presented multiple or multifocal skin tumors, thus, the proportion of SCC and BCC for each of the included study was calculated using the total number of cancer lesions reported in that study as the denominator. Then, using the restricted maximum-likelihood estimator of the random effect model, we calculated the weighted average proportions for the two keratinocyte carcinomas after transformation of the raw proportions by the Freeman–Turkey double arcsine method in order to achieve normality and variance stability [20]. Heterogeneity across included studies was assessed using I^2^, *τ*^2^, and Cochrane *Q* test. I^2^ values below 25% were considered low heterogeneity; 25–75%, moderate heterogeneity; above 75%, high heterogeneity. Cochran *p* values below 0.1 were considered signiﬁcant. Publication bias was assessed by the funnel plot and Egger's unweighted regression test.

## 3. Result

### 3.1. Study Selection

**Figure 1:**
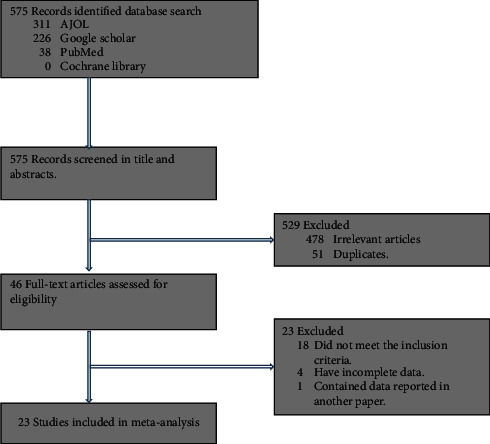
PRISMA flow chart of the article selection process.

Database search yielded 575 potentially relevant records, most of which were irrelevant articles that were excluded following title and abstract screening. Forty-six full-text articles were acquired and assessed for eligibility of which 23 fulfilled the inclusion criteria (Figure 1).

### 3.2. Study Characteristics

Characteristics of the 23 included studies are shown in Table 2. The studies were 23 case series and cross-sectional studies with the publication year ranging from 1953 to 2020. Most of the studies were done in Nigeria followed by Tanzania. Specifically, 10 of the studies [15, 21–29] had only albino skin cancer subjects; 4 studies [16, 30–32] equally had only albino subjects but reported on skin cancers and other skin diseases; 9 studies [13, 33–40] had mixed samples of albinos and nonalbinos.

The risk of bias assessment or methodological quality score for the included studies is presented in Table 3. The study quality score ranged from 3 to 6 by the assessment tool we used. Twenty-one studies were assessed to be of high quality and 2 were assessed low quality.

### 3.3. Synthesis of Results

From the 23 studies, we identified 540 African albinos presenting 695 histologically confirmed skin cancers. These were composed of 274 males and 241 females with sex missing in 24 cases. There were 419 SCC and 249 BCC among the 695 cancer lesions (Table 4).

**Figure 2:**
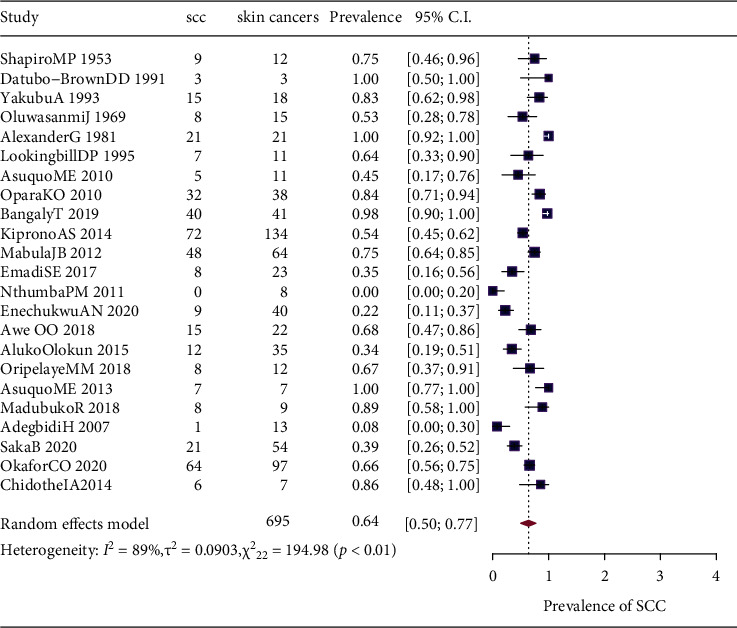
Forest plot showing the pooled prevalence of squamous cell carcinoma in 23 included studies.

By random effect meta-analysis, the pooled prevalence of SCC for the 23 studies was 64% (95CI; 50–77%). For BCC, the pooled prevalence was 31% (95CI; 19–41%).  Figure 2 is a forest plot showing the individual study prevalence of SCC, the pooled prevalence, and the heterogeneity statistics.

**Figure 3:**
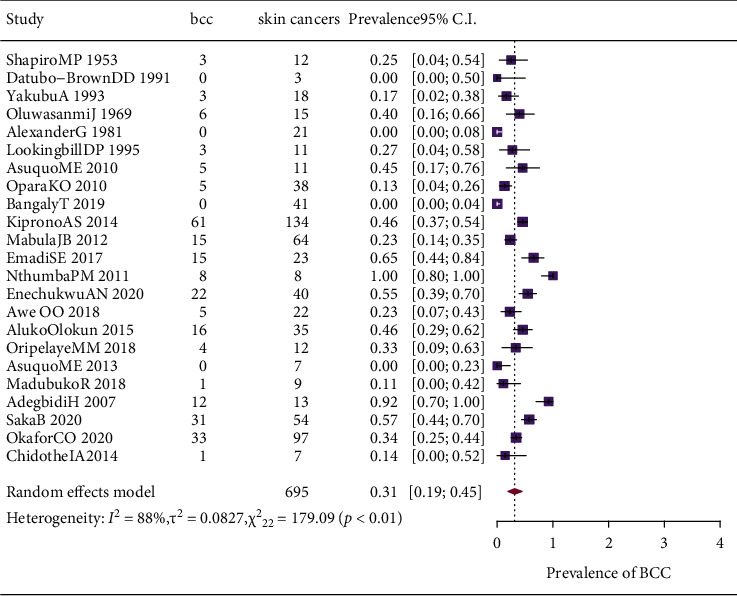
Forest plot showing the pooled prevalence of basal cell carcinoma.

Individual study prevalence and pooled prevalence of BCC are similarly displayed in Figure 3.There was high heterogeneity in the prevalence estimates across all the included studies (Cochrane *Q* (df _22_) = 195, *p*=<0.01). Also, I^2^ the ratio of between the study variance to the total variance was 89% (95% CI; 85%–96%) just as *τ*^2^; another measure of variance between studies was 0.1(95%CI; 0.05–0.2), further highlighting the heterogeneity of the prevalence estimates across studies.

Sensitivity analysis did not significantly alter the pooled estimate or the heterogeneity statistics. Also, moderator analyses were done using a sample size greater than 20, country of study (Nigeria vs. others), and study specifying multifocal tumour as moderating variables. Observed heterogeneity was not explained by any of the moderating variables as *R*^2^, and The amount of heterogeneity accounted for by the moderators was 0%.

**Figure 4:**
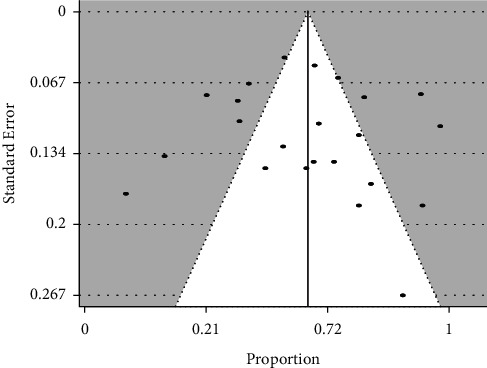
Funnel plot of the included studies.

Publication bias: Egger's test of funnel plot asymmetry was not significant, *z* = 0.60, *p*=0.55, suggesting a lack of publication bias in the present review (Figure 4).

## 4. Discussion

African albinos have creamy white skin, sandy yellow hair, and brown hazel eyes, which are the phenotypic consequence of inherited genetic defects in melanin synthesis and pigmentation of their skin, hair, and ocular tissues [41]. This genetic inheritance and distinctive physical appearance in a population of black pigmented people predisposes the African albino to some existential challenges such as social discrimination, and in some places, physical assault with body dismemberment [42, 43]. Healthwise, they uniformly develop visual abnormalities and have elevated risk for photodermatosis and skin cancer [10, 30].

For the African albino, however, skin cancer is a very important health problem. Being deficient in the protective melanin pigment and inhabiting a climate of high ambient sunshine predisposes African albinos to the photocarcinogenic effect of high UV radiation. Epidemiologic studies of skin cancer in Africans report that, compared with normally pigmented Africans, African albinos have higher frequency of keratinocyte cancers, which occur at a significantly younger age, and they develop multiple and/or recurrent lesions which feature more at sun-exposed body sites [25].

Meta-analysis, originally applied in the synthesizing results of clinical trials and determining the effects of treatment interventions, has found escalating use in deriving precise estimates of disease frequency such as incidence rate and prevalence proportions [44].

The present systematic review synthesized data from eligible case series and cross-sectional studies of skin cancers in African albinos and attempted to establish the prevalence proportion of SCC and BCC using the methods of meta-analysis.

Elaborating on prevalence as a variable, Barendregt et al. [44] states that disease prevalence is a proportion which is derived by dividing the number of cases of the disease in a population by the population number. Its value always lies between 0 and 1, and sum over multicategories amounts to 1 [44]. A very notable feature of skin cancer in African albinos is the propensity for multifocal or multiple tumours with an individual patient sometimes presenting histologically different cancer types. Thus, the number of skin cancer lesions we abstracted (695) was more than the number of albinos with skin cancer (540). We determined the prevalence or proportion of SCC and BCC in all the histologically confirmed skin cancer lesions of African albinos.

This pooled prevalence of SCC was 64% while that of BCC was 31%, and these represents average proportion of SCC and BCC in all the studies weighted by the inverse of their sampling variances. Heterogeneity statistics indicate a lack of homogeneity in the reported proportions across all the included studies. This was not explainable by sensitivity or moderator analysis. Small sample sizes and variations in study settings could be responsible. In spite of this observed heterogeneity, the estimated prevalence figures appear valid. This is because, by crude unweighted pooling of the individual study proportions (analogous to the ditched “vote counting” method previously used in meta-analysis of clinical trials and interventions), the proportion of SCC among all histologically confirmed skin cancer lesions in African albinos would be 60.2% (419/695) and that of BCC, 35.8% (249/695). These figures are quite comparable to the pooled prevalence established by meta-analyses.

Marçon et al. [45] in Brazil suggest that the frequency of BCC might be equal to SCC in albinos and that studies reporting more SCC are hospital excision biopsies of advanced tumours in which SCCs are more likely to predominate, being the more aggressive of the two tumours and often requiring surgical attention. This view seems to be supported by the fact that two studies that reported more BCC than SCC in African albinos feature biopsies taken at routine dermatological examination and surveillance programmes [16, 17].

Publication bias has been explained, chiefly, in terms of preferential publication of manuscripts with statistically significant results to the exclusion of those with nonsignificant results. But some other study characteristics such as funding source, research setting, and prevailing theories at the time of publication have been found to equally influence publication [46]. Even though publication bias could confound systematic reviews, it has been questioned if the traditional methods employed in the assessment of publication bias for comparative studies are appropriate for observational studies of the type used in meta-analysis of proportions. The studies examined in meta-analysis of proportion, being noncomparative, are not subject to considerations of statistical significance and the direction of result, which are known to preferentially influence publication of clinical trials [19, 46, 47]. Nevertheless, we assessed for publication bias using the funnel plot. Egger's test of funnel plot asymmetry was nonsignificant, suggesting a lack of publication bias in the published studies.

Our study has some strengths and limitations. First, it is, to the best of our knowledge, the first meta-analysis on skin cancer in African albinos and thus represents the largest study of albinos with skin cancer to date. The study was able to yield data, which bolstered the previously reported epidemiologic trends of skin cancer and statistically established that the prevalence for SCC and BCC in skin cancer lesions. The study is limited by the small sample sizes of the available studies and dearth of publications devoted exclusively to the subject of albino skin cancer. In addition, because our study is a review, we could not validate the histological types of the skin cancers reported by the included studies.

## 5. Conclusion

In conclusion, we estimated the prevalence proportion of SCC and BCC in histologically confirmed skin cancer lesions in African albinos. The pooled average prevalence proportion of SCC amongst all histologically confirmed skin cancer lesions in African albinos was 64% (95% CI; 50–77%) and the prevalence of BCC was 31% (95% CI; 19–45%). Squamous cell carcinoma is the predominant type of keratinocyte carcinoma reported in African albinos overall. This predominance of keratinocyte carcinomas in African albinos relates more the with pattern of occurrence in pigmented Africans rather than Caucasians.

## Figures and Tables

**Table 1:** Risk of bias assessment tool [19]].

**A. Selection** (Does the patient(s) represent(s) the whole experience of the investigator (center) or is the selection method unclear to the extent that other patients with similar presentation may not have been reported?)
i. Cases were selected over a specified range of period? 2 points
ii. Selection approach unclear? 1 point
**B. Ascertainment of outcome:** (How were the cases ascertained?)
i. Clinical records? 2 points
ii. Self-report or other methods? 1point
**C. Reporting:**
i. Cases described in sufficient details? 2 points
ii. Cases scanty with some missing information? 1point

**Table 2:** Included studies, country of study, number of albinos, and skin cancer type.

Author; year	Country	No. of albinos with skin cancer	Skin cancer type^*∗*^					Males	Females
			SCC	BCC	CM	Others	Total		
Shapiro MP; 1953	South Africa	12	9	3	0	0	12	8	4
Datubo–Brown DD; 1991	Nigeria	3	3	0	0	0	3	0	0
Yakubu A; 1993	Nigeria	18	15	3	0	0	18	12	6
Oluwasanmi J; 1969	Nigeria	15	8	6	0	1	15	12	3
Alexander G; 1981	Tanzania	10	21	0	0	0	21	9	1
Lookingbill DP; 1995	Tanzania	10	7	3	0	1	11	5	5
Asuquo ME; 2010	Nigeria	9	5	5	1	0	11	5	4
OparaK O; 2010	Nigeria	20	32	5	0	1	38	10	10
Bangaly T; 2019	Guinea	30	40	0	0	1	41	12	18
Kiprono AS; 2014	Tanzania	86	72	61	1	0	134	41	45
Mabula JB; 2012	Tanzania	64	48	15	1	0	64	38	26
Emadi SE; 2017	Kenya	20	8	15	0	0	23	13	7
Nthumba PM; 2011	Kenya	8	0	8	0	0	8	5	3
Enechukwu AN; 2020	Nigeria	18	9	22	0	9	40	9	9
Awe OO; 2018	Nigeria	22	15	5	2	0	22	11	11
Aluko Olokun; 2015	Nigeria	35	12	16	0	7	35	17	18
Oripelaye MM; 2018	Nigeria	12	8	4	0	0	12	0	0
Asuquo ME; 2013	Nigeria	4	7	0	0	0	7	2	2
Madubuko R; 2018	Nigeria	9	8	1	0	0	9	0	0
Adegbidi H; 2007	Benin Republic	5	1	12	0	0	13	3	2
Saka B; 2020	Togo	33	21	31	0	2	54	17	16
Okafor CO; 2020	Nigeria	86	64	33	0	0	97	38	48
Chidothe IA; 2014	Malawi	7	6	1	0	0	7	6	1

^*∗*^SCC: squamous cell carcinoma; BCC: basal cell carcinoma; CM: cutaneous melanoma.

**Table 3:** Risk of bias assessment score for each of the included study.

	1st Author; study year	A	B	C	Aggregate score^*∗*^
		Selection	Ascertainment of outcome	Reporting	
1	Adegbidi H; 2007	2	2	2	6
2	Alexander G; 1981	1	2	2	5
3	Aluko Olokun; 2015	1	1	2	4
4	Asuquo ME; 2013	1	2	2	5
5	Asuquo, ME; 2010	2	2	2	6
6	Awe OO; 2018	2	2	1	5
7	Bangaly T; 2019	2	2	2	6
8	Chidothe IA; 2014	1	2	2	5
9	Datubo–Brown DD; 1991	1	2	1	4
10	Emadi SE; 2017	2	2	2	6
11	Enechukwu AN; 2020	2	2	1	5
12	Kiprono AS; 2014	2	2	2	6
13	Lookingbill DP; 1995	2	2	1	5
14	Mabula JB; 2012	2	2	2	6
15	Madubuko R; 2018	2	2	2	6
16	Nthumba PM; 2011	1	2	2	5
17	Okafor CO; 2O2O	2	2	2	6
18	Oluwasanmi J, 1969	2	2	2	6
19	Opara KO; 2010	2	2	2	6
20	Oripelaye MM; 2018	1	2	2	5
21	Saka B; 2020	2	2	2	6
22	Shapiro MP, 1953	2	2	2	6
23	Yakubu A, 1993	2	2	2	6

^*∗*^Aggregate score: 3, 4 (low quality); 5, 6 (high quality).

**Table 4:** Skin cancer type and sex of 540 African albinos with cutaneous malignancy^*∗*^.

Variable	Frequency
Sex	
Male	275
Female	241
Missing	24
Total	540
Skin cancer type	419
Squamous cell carcinoma	419
Basal cell carcinoma	249
Cutaneous melanoma	5
Others^*∗∗*^	22
Total	695

;^*∗*^Some patients presented multifocal cancers: ^*∗∗*^ 9 basosquamous carcinoma, 10 adenoid cystic carcinoma, 1 malignant adnexal tumour, 1 sarcoma, and 1 unspecified histology.

## Data Availability

The data used to support the findings of this study are included within the article.

## References

[1] Perera E., Gnaneswaran N., Staines C., Win A. K., Sinclair R. (2015). Incidence and prevalence of non-melanoma skin cancer in Australia: a systematic review. *Australasian Journal of Dermatology*.

[2] Rogers H. W., Weinstock M. A., Feldman S. R., Coldiron B. M. (2015). Incidence estimate of nonmelanoma skin cancer (keratinocyte carcinomas) in the US population, 2012. *JAMADermatol*.

[3] Buettner P. G., Raasch B. A. (1998). Incidence rates of skin cancer in Townsville, Australia. *International Journal of Cancer*.

[4] Venables Z. C., Nijsten T., Wong K. F. (2019). Epidemiology of basal and cutaneous squamous cell carcinoma in the U.K. 2013–15: a cohort study. *British Journal of Dermatology*.

[5] Gloster H. M., Neal K. (2006). Skin cancer in skin of color. *Journal of the American Academy of Dermatology*.

[6] Agbai O. N., Buster K., Sanchez M. (2014). Skin cancer and photoprotection in people of color: a review and recommendations for physicians and the public. *Journal of the American Academy of Dermatology*.

[7] Oseni G. O., Olaitan P. B., Komolafe A. O., Olaofe O. O., Akinyemi H. A. M., Suleiman O. A. (2015). Malignant skin lesions in Oshogbo, Nigeria. *The Pan African medical journal*.

[8] Ochicha O., Edino S. T., Mohammed A. Z., Umar A. B. (2004). Dermatological malignancies in Kano Northern Nigeria: a histopathological review. *Annals of African Medicine*.

[9] Chalya P. L., Gilyoma J. M., Kanumba E. S. (2012). Dermatological malignancies at a University Teaching Hospital in north-western Tanzania: a retrospective review of 154 cases. *Tanzania Journal of Health Research*.

[10] Hong E. S., Zeeb H., Repacholi M. H. (2006). Albinism in Africa as a public health issue. *BMC Public Health*.

[11] Raasch B. A., Buettner P. G. (2002). Multiple nonmelanoma skin cancer in an exposed Australian population. *International Journal of Dermatology*.

[12] Chahal H. S., Rieger K. E., Sarin K. Y. (2017). Incidence ratio of basal cell carcinoma to squamous cell carcinoma equalizes with age. *Journal of the American Academy of Dermatology*.

[13] Okafor O. C., Onyishi N. T. (2021). Primary cutaneous malignancies in non-albino and albino Africans. *International Journal of Dermatology*.

[14] Nthumba P. M. (2010). Marjolin’s ulcers in sub-Saharan Africa. *World Journal of Surgery*.

[15] Mabula J. B., Chalya P. L., Mchembe M. D. (2012). Skin cancers among Albinos at a University teaching hospital in Northwestern Tanzania: a retrospective review of 64 cases. *BMC Dermatology*.

[16] Enechukwu N. A., Ogun G. O., Ezejiofor O. I. (2020). Histopathologic patterns of cutaneous malignancies in individuals with oculocutaneous albinism in Anambra state, Nigeria: a paradigm swing?. *Ecancermedicalscience*.

[17] Saka B., Teclessou J. N., Akakpo S. A. (2020). A histopathological study of skin lesions in individuals with oculocutaneous albinism in Togo in 2019. *Journal of Skin Cancer*.

[18] Onyishi N. T., Ohayi S. R. (2021). Cutaneous melanoma in African albinos: a systematic review. *Journal of Clinical and Diagnostic Research*.

[19] Murad M. H., Sultan S., Haffar S., Bazerbachi F. (2018). Methodological quality and synthesis of case series and case reports. *BMJ Evidence-Based Medicine*.

[20] Wang N. (2018). *How to Conduct a Meta-Analysis of Proportions in R: A Comprehensive Tutorial Conducting Meta-Analyses of Proportions in R*.

[21] Alexander G. A., Henschke U. K. (1981). Advanced skin cancer in Tanzanian albinos:Preliminary observations. *Journal of the National Medical Association*.

[22] Asuquo M. E., Otei O. O., Omotoso J., Bassey E. E. (2010). Letter: skin cancer in albinos at the university of calabar teaching hospital, calabar, Nigeria. *Dermatology Online Journal*.

[23] Opara K. O., Jiburum B. C. (2010). Skin cancers in albinos in a teaching Hospital in eastern Nigeria - presentation and challenges of care. *World Journal of Surgical Oncology*.

[24] Traore B., Barry A., Kourouma T., Keita M., Cisse M. (2019). Skin cancers in albinos at surgical oncology unit of donka national hospital (conakry). *Cancer Studies and Therapeutics*.

[25] Kiprono S. K., Chaula B. M., Beltraminelli H. (2014). Histological review of skin cancers in African Albinos: a 10-year retrospective review. *BMC Cancer*.

[26] Awe O. O., Azeke T. A. (2018). Cutaneous cancers in Nigerian albinos: a review of 22 cases. *Nigerian Journal of Surgery*.

[27] Asuquo M. E., Otei O. O., Bassey I., Ebughe G. (2013). Oculocutaneous albinism and skin cancer in Calabar. *International Journal of Medicine and Medical Sciences*.

[28] Saka B., Akakpo S. A., Teclessou J. N. (2019). Skin cancers in people with albinism in Togo in 2019: results of two rounds of national mobile skin care clinics (in review). *BMC Dermatology*.

[29] Chidothe I. A., Masamba L. (2014). Neoadjuvant chemotherapy in albinos with locally advanced skin cancer at a blantyre hospital: case series. *Malawi Medical Journal*.

[30] Lookingbill D. P., Lookingbill G. L., Leppard B. (1995). Actinic damage and skin cancer in albinos in northern Tanzania: findings in 164 patients enrolled in an outreach skin care program. *Journal of the American Academy of Dermatology*.

[31] Emadi S. E., Suleh A. J., Babamahmoodi F. (2017). Common malignant cutaneous conditions among albinos in Kenya. *Medical Journal of the Islamic Republic of Iran*.

[32] Roli M. C., Abel O. (2018). Photodermatoses in the Nigerian albino: a study in an urban hospital in southern Nigeria. *Journal of Medicine in the Tropics*.

[33] Shapiro M. P., Keen P., Cohen L., Murray J. F. (1953). Skin cancer in the South African Bantu. *British Journal of Cancer*.

[34] Datubo-Brown D. D. (1991). Primary malignant skin tumor in Nigerians. *Journal of the National Medical Association*.

[35] Yakubu A., Mabogunje O. A. (1993). Skin cancer in African albinos. *Acta Oncologica*.

[36] Oluwasanmi J. O., Williams A. O., Alli A. F. (1969). Superficial cancer in Nigeria. *British Journal of Cancer*.

[37] Nthumba P. M., Cavadas P. C., Landin L. (2011). Primary cutaneous malignancies in sub-Saharan Africa. *Annals of Plastic Surgery*.

[38] Aluko-Olokun B., Olaitan A. A. (2015). Skin cancer risk factor reduction in Africa: assessment of use of antiretroviral therapy services by human immunodeficiency virus positive albinos. *HIV & AIDS Review*.

[39] Oripelaye M. M., Oladele A. O., Olanrewaju F. O., Olaofe O. O. (2018). The evolving pattern of primary skin cancers in ile-ife, Nigeria. *Serbian Journal of Dermatology and Venereology*.

[40] Adegbidi H., Yedomon H., Atadokpede F., Balley-Pognon M.-C., Do Ango-Padonou F. (2007). Skin cancers at the national university hospital of cotonou from 1985 to 2004. *International Journal of Dermatology*.

[41] Kromberg J. G. R., Bothwell J., Kidson S. H., Manga P., Kerr R., Jenkins T. (2012). Types of albinism in the black southern Africa population. *East African Medical Journal*.

[42] Taylor J., Bradbury-Jones C., Lund P. (2019). Witchcraft-related Abuse and murder of children with albinism in sub-saharan Africa: a conceptual review. *Child Abuse Review*.

[43] Brilliant M. H. (2015). Albinism in Africa: a medical and social emergency. *International Health*.

[44] Barendregt J. J., Doi S. A., Lee Y. Y., Norman R. E., Vos T. (2013). Meta-analysis of prevalence. *Journal of Epidemiology & Community Health*.

[45] Marçon C. R., Moraes J. C., de Olivas Ferreira M. A. M., Oliari C. B. (2020). Dermatological and epidemiological profiles of patients with albinism in são paulo, Brazil, between 2010 and 2017: a cross-sectional study. *Dermatology*.

[46] Coburn K. M., Vevea J. L. (2015). Publication bias as a function of study characteristics. *Psychological Methods*.

[47] Maulik P. K., Mascarenhas M. N., Mathers C. D., Dua T., Saxena S. (2011). Prevalence of intellectual disability: a meta-analysis of population-based studies. *Research in Developmental Disabilities*.

